# Letter to the editor regarding “Posterior intra-articular fixation stabilizes both primary and secondary sacroiliac joints: a cadaveric study and comparison to lateral trans-articular fixation literature”

**DOI:** 10.1186/s13018-023-04048-1

**Published:** 2023-08-03

**Authors:** Derek P. Lindsey, Scott A. Yerby

**Affiliations:** SI-BONE, Santa Clara, CA USA

We read the recent article by Sayed et al. [1] with interest. The authors report the results from a cadaveric biomechanics study that evaluated sacroiliac (SI) joint motion after placement of cortical allograft into the SI joint using a posterior approach under unilateral and bilateral conditions. The study was performed using a multi-directional flexibility model with range of motion tracked during application of pure moments. This data, which was previously published from Sayed et al. [2], was compared to a published study by Lindsey et al. [3] that evaluated SI joint motion after placement of lateral transfixing triangular titanium implants under unilateral and bilateral conditions. The authors conclude that the use of cortical allograft from the posterior approach provides equivalent stabilization in flexion–extension and superior stabilization in lateral bending and axial rotation when compared with a lateral approach.

As the authors of the comparative study (Lindsey et al*.*), we applaud Sayed et al*.* for using the same testing methodology. We would like to highlight some key differences between the studies, including methodological issues, that call the comparative results into question.

## Specimen demographics

The three specimens in Sayed et al*.* are relatively young (34–37 years old) with exceptionally good bone quality (L1–L4 *t*-score = 0.8 ± 0.2, L4 bone density = 1.3 ± 0.2 g/cm^2^). Although the ratio of female to male (2:1) is consistent between the compared studies, the age is considerably lower (average 35 years) compared with Lindsey et al*.* (average 47 years). In addition, the age of the tested specimens is substantially lower than that reported for patients (average 68 years) treated with this cortical allograft [4]. Based upon the known relationship between increasing age and decreasing bone density, it is unclear that the results observed in these younger, high bone quality specimens are comparable to those from Lindsey et al*.* or applicable to the intended patient population.

## Minimal sample size

Sayed et al*.* only used a total of 3 cadaveric pelvises (as described in Sayed et al. [2]) that are described herein as 6 cadaveric SI joints (noted as four females and two males). The calculated sample size references the standard deviation, significance, power, and effect size from Soriano-Baron et al. [5] while using a considerably larger effect size (50% vs 30%); the resultant sample size of four SI joints is drastically less than the 7 pairs (i.e., 14 SI joints) used by Soriano-Baron et al. Although sample sizes for cadaveric studies are often limited by specimen availability, the use of only 3 pelvis specimens is substantially smaller than the number used for the lateral implantation study by Lindsey et al*.* (8 pelvises) and well outside the norms of in vitro studies.

## Unsupported data pooling

As a result of the decision to use 3 cadaveric specimens, the authors resort to pooling data from the primary and secondary SI joints to meet the minimal sample size of 4 joints. The result is that data from the unilaterally *treated* joints were combined with the secondary *untreated* joint to generate the pooled unilateral data. This presents multiple methodological and statistical issues. The authors do not present a statistical rationale to pool their data other than the statement “… no significant differences were observed in our samples, between primary and secondary”. However, an analysis of the individual specimen unilateral data from Sayed et al. [2] with a paired *t* test on the ROM data from the right and left joints of the 3 specimens results in a minimum *P* value < 0.2, which is suggestive that the data are not comparable. Further statistical analysis would be required to determine if this data is equivalent (i.e., the same) or not significantly different to allow for pooling [6]. Unfortunately, the authors did not test enough samples to meet their predefined sample size nor properly analyze their data to make a definitive statement on whether the groups are equivalent and justify pooling data for comparison.

## Varied cortical allograft placement

The authors describe fixation being performed by a board-certified interventional pain specialist following the manufacturer’s technique. Upon closer inspection of the presented images, there is substantial difference in the placement between the three specimens. As shown in the radiographic imaging, there is significant difference in the cephalad implant trajectory shown in Fig. 3 of Sayed et al. [2] and the posterior to anterior trajectory shown in Figs. 2 and 5 of Sayed et al. [1]. As a result, it is unclear what impact the variation in implant trajectory has on the large variations in SI joint ROM reduction reported for Specimens A, B, and C in Sayed et al. [2]. Based upon the individual specimen results, it appears that one trajectory may offer substantially more stabilization than the other; this is unclear, however, based on the inadequate sample size. For a paper that is focused on comparing trajectories, it is perplexing that the authors chose to ignore the potential impact of implant trajectory for the cortical allografts.

## Clinical evidence

Lastly, Sayed et al. [1] selectively referenced postoperative complication rates of 11–32.5% for the lateral approach to SI joint fusion. In a pooled analysis of prospective trials, complication rates from lateral transiliac SI joint fusion using triangular titanium implants were smaller [7]. Of 326 subjects who underwent SI joint fusion, early surgical revisions due to malposition occurred in 1.2% of patients, late revision surgery occurred in 2.9%, and wound problems (including any wound problem requiring antibiotics or surgical management) occurred in 2.5%. Although complication rates can be variable, inclusion of accurate complication rates (including those from Level I studies) is vital to allow for critical assessment.

In summary, although the authors imply that the same methodology was used between the current study and in the evaluation of the lateral transfixing implant, there remain numerous substantial methodological flaws regarding specimen demographics, sample size, data pooling, and cortical allograft placement that call the conclusions into question and prevent the reader from making a clear and fair assessment between the treatments.

## Data Availability

Not applicable.

## References

[1] Sayed D, Amirdelfan K, Hunter C, Raji OR (2023). Posterior intra-articular fixation stabilizes both primary and secondary sacroiliac joints: a cadaveric study and comparison to lateral trans-articular fixation literature. J Orthop Surg Res.

[2] Sayed D, Amirdelfan K, Naidu RK, Raji OR, Falowski S (2021). A cadaver-based biomechanical evaluation of a novel posterior approach to sacroiliac joint fusion: analysis of the fixation and center of the instantaneous axis of rotation. Med Devices Auckl NZ.

[3] Lindsey DP, Parrish R, Gundanna M, Leasure J, Yerby SA, Kondrashov D (2018). Biomechanics of unilateral and bilateral sacroiliac joint stabilization: laboratory investigation. J Neurosurg Spine.

[4] Sayed D, Balter K, Pyles S, Lam CM (2021). A multicenter retrospective analysis of the long-term efficacy and safety of a novel posterior sacroiliac fusion device. J Pain Res.

[5] Soriano-Baron H, Lindsey DP, Rodriguez-Martinez N, Reyes PM, Newcomb A, Yerby SA (2015). The effect of implant placement on sacroiliac joint range of motion: posterior vs trans-articular. Spine.

[6] Harris AHS, Fernandes-Taylor S, Giori N (2012). “Not statistically different” does not necessarily mean “the same”: the important but underappreciated distinction between difference and equivalence studies. J Bone Joint Surg Am.

[7] Dengler J, Duhon B, Whang P, Frank C, Glaser J, Sturesson B (2017). Predictors of outcome in conservative and minimally invasive surgical management of pain originating from the sacroiliac joint: a pooled analysis. Spine.

